# MACC1 revisited – an in-depth review of a master of metastasis

**DOI:** 10.1186/s40364-024-00689-4

**Published:** 2024-11-23

**Authors:** Paul Curtis Schöpe, Sebastian Torke, Dennis Kobelt, Benedikt Kortüm, Christoph Treese, Malti Dumbani, Nazli Güllü, Wolfgang Walther, Ulrike Stein

**Affiliations:** 1 Experimental and Clinical Research Center, Charité - Universitätsmedizin Berlin and Max-Delbrück-Center for Molecular Medicine, Berlin, Germany; 2 German Cancer Consortium (DKTK), Berlin and German Cancer Research Center (DKFZ), Heidelberg, Germany

**Keywords:** MACC1, Biomarker, Metastasis, Cancer

## Abstract

Cancer metastasis remains the most lethal characteristic of tumors mediating the majority of cancer-related deaths. Identifying key molecules responsible for metastasis, understanding their biological functions and therapeutically targeting these molecules is therefore of tremendous value. Metastasis Associated in Colon Cancer 1 (MACC1), a gene first described in 2009, is such a key driver of metastatic processes, initiating cellular proliferation, migration, invasion, and metastasis in vitro and in vivo. Since its discovery, the value of MACC1 as a prognostic biomarker has been confirmed in over 20 cancer entities. Additionally, several therapeutic strategies targeting MACC1 and its pro-metastatic functions have been developed. In this review, we will provide a comprehensive overview on MACC1, from its clinical relevance, towards its structure and role in signaling cascades as well as molecular networks. We will highlight specific biological consequences of MACC1 expression, such as an increase in stem cell properties, its immune-modulatory effects and induced therapy resistance. Lastly, we will explore various strategies interfering with MACC1 expression and/or its functions. Conclusively, this review underlines the importance of understanding the role of individual molecules in mediating metastasis.

## Introduction

Cancer metastasis is the most lethal attribute of cancer and directly linked to patient survival, which is about 80% in early, non-metastasized stages, but below 10%, when distant metastases have formed. Conclusively, metastasis is responsible for the majority of cancer deaths [1, 2]. As exemplified for colorectal cancer (CRC), currently about 2 million new cases are reported worldwide each year, which is prognosticated to be increased to 3 million in 2040. This is currently associated with one million of cancer deaths, which will be increased to more than 1.5 million in 2040 [3]. Despite the improvements made for solid cancer treatment, metastasis-targeting therapies still lack behind. Consequently, metastasis formation presents a major therapeutic challenge, critically limiting successful therapy. Therefore, it is an important and urgent clinical need to better understand metastatic processes to more successfully combat cancer metastasis. To identify cancer patients at high risk for metastasis, biomarkers can be employed, which in particular act as key drivers for metastasis and serve as therapeutic targets—a concept, which is gaining worldwide importance for clinical interventions.

**Fig. 1:**
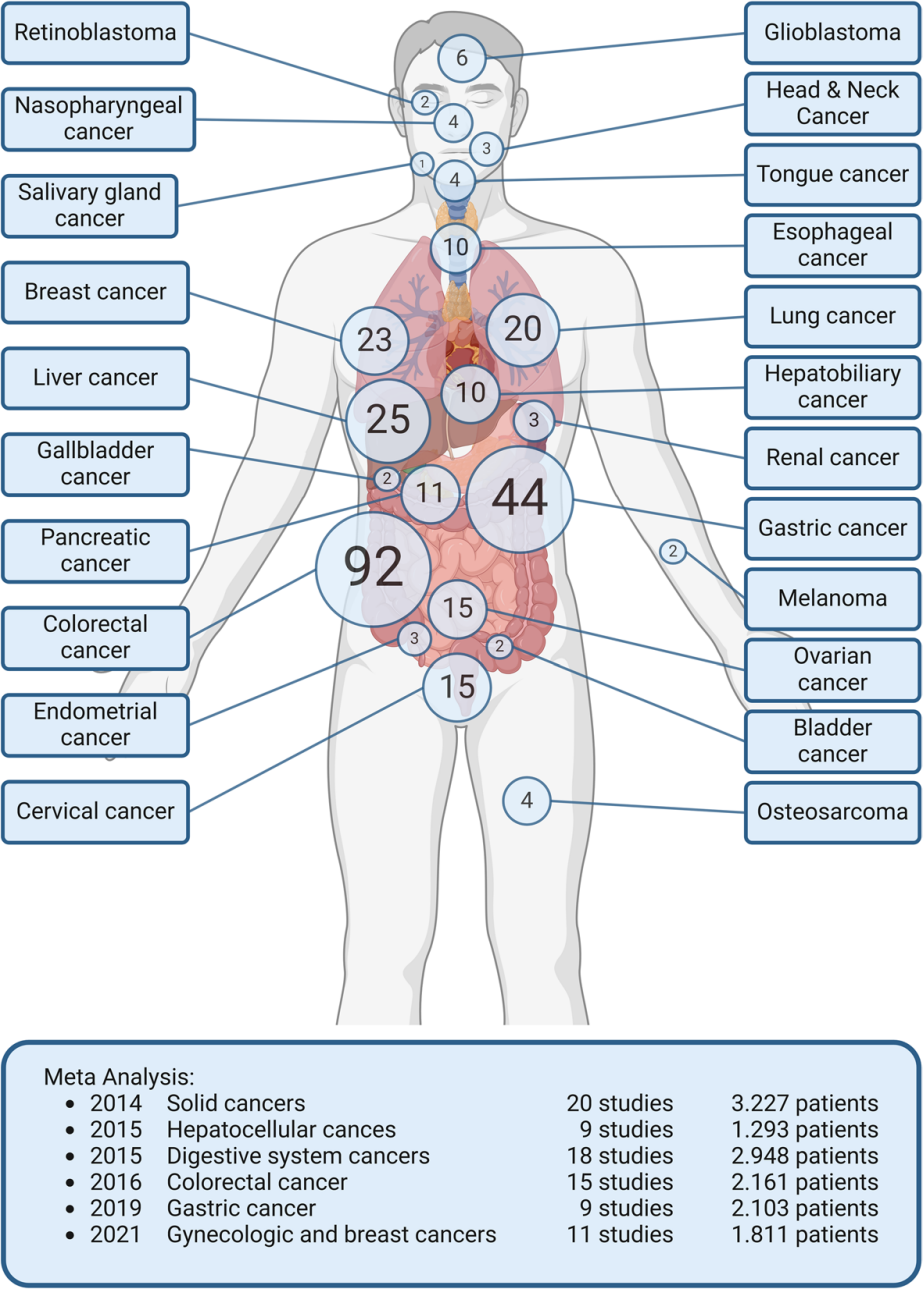
Overview on publications regarding MACC1 in various cancer entities. The role of MACC1 has been investigated in more than 20 cancer types. The size of the circles indicate the number of studies performed in the listed cancer entities. The box lists MACC1 meta-analyses and their respective total patient numbers. Updated from [5].Created with BioRender

In search of new, causal drivers of metastasis the gene MACC1, a previously undescribed gene, was identified in tumor tissue from CRC patients in 2009 [4]. Since then, MACC1 has been established as a valuable biomarker for metastasis and survival prognosis as well as for therapy response in more than 20 distinct solid cancer entities [5]. Meanwhile, about 350 succession papers (in PubMed alone) from research groups worldwide were published to date (Fig. 1), including meta-analyses of hepatocellular, gastrointestinal, colorectal, gastric, gynecological and breast cancer. MACC1 levels, quantified in the primary tumors or patient blood, are significantly higher in those cancers that develop distant metastases compared to those which do not. Based on this, a personalized prognosis can be made, whether a patient will develop metastasis (or not) before the event of clinically detectable metastasis. Combination of MACC1 with further biomarkers within the MACC1 network significantly improves this prognostication.

As key biological functions, MACC1 induces fundamental metastasis-associated phenotypes in vitro, such as migration, invasion, cell dissemination, and proliferation, contributes to wound healing, colony formation, anti-apoptosis, and inflammation. Moreover, it is decisively involved in stemness, protein trafficking/clathrin-mediated endocytosis (CME), in biomechanics and contributes to the development of chemoresistance. In vivo, MACC1 impacts tumor initiation and progression as well as liver and lung metastases in colon cancer xenografted (CDX, PDX) and MACC1 transgenic mouse models [4, 6, 7]. MACC1 regulation mechanisms were reported at the transcriptional, post-transcriptional, translational and post-translational level. Transcriptional targets and protein–protein interactors of MACC1 were unveiled as new diagnostic, prognostic and predictive key players for tumor progression and metastasis.

The identification and characterization of whole MACC1 networks—MACC1 together with regulating RNAs and/or proteins—has inspired the development of novel therapeutic approaches, which are currently tested in clinical trials as specific tailored approaches to intervene in metastasis formation. In this context, newly identified or repositioned small molecule inhibitors acting on MACC1 and its network are investigated. Taken together, MACC1 and associated networks are valuable tools for cancer diagnosis, prognosis, and prediction. This MACC1 review will summarize recent developments in detail from this field of cancer/metastasis research and clinical care focusing on the great potential to combat metastatic disease for improved patient outcome.

## Clinical relevance of MACC1 as strong prognostic and predictive biomarker

Since the first description of MACC1, its value and importance as a biomarker have been strongly consolidated [4]. In this context, next to publications regarding the pathophysiology of this protein, numerous papers have been published on the clinical relevance of MACC1 as a prognostic and predictive biomarker in solid cancers. The majority of these studies focused on CRC, however, the role of MACC1 has been studied in over 20 solid cancer entities including common cancers such as lung, breast and gastric cancer. Surprisingly, no literature was found focusing on MACC1 in prostate cancer, the second most common cancer in males, pointing to the necessity of exploring the role of MACC1 in this entity. In the initial description of MACC1, its strong potential as a negative prognostic and predictive biomarker was demonstrated based on a small cohort of 60 patients [4]. In the following years, several further publications and subsequently a meta-analysis of 10 studies and 1500 patients confirmed these results for CRC patients [8–10].

Expanding beyond CRC, MACC1 has also been shown to predict poor survival and metastasis formation in other biliodigestive cancers, including esophageal, gastric, cholangial, gallbladder, pancreatic and hepatocellular carcinomas [11–16]. It was demonstrated that in esophageal squamous cell carcinoma (SCC) MACC1 expression correlated positively with the expression of Snail, which has been associated with tumor epithelial-mesenchymal transition (EMT) and less favorable, more advanced tumor stages, lymph node metastasis (LNM) and Tumor Nodes Metastasis (TNM) stages [16]. Furthermore, in 2022 the prognostic value of MACC1 in esophageal and gastric adenocarcinomas was shown for the first time in a Caucasian patient cohort. In this study, a subgroup analysis of tumors with low risk morphology (V0 and L0) showed that high expressing MACC1 tumors elicit a significant reduction in overall survival (OS) (V0: MACC1-low: 115.7 months vs MACC1-high: 65.4 months; L0: MACC1-low 134.9 months vs MACC1-high 80.9 months), highlighting that MACC1 is of particular interest in a situation when patients have no observable vascular or lymphatic invasion and biomarkers would aid in predicting the further disease course [15]. Interestingly, an elevated MACC1 expression was detected in patients with a history of tumor recurrence in another study focusing on intrahepatic and hilar cholangiocarcinoma patients, confirming that MACC1 can be used as an independent prognostic biomarker for the OS of Klatskin tumor patients [12]. For gallbladder cancer, Chen et al. identified that high MACC1 expression was linked to LNM and perineural invasion but was not associated with a history of gallstones or the histological grade [13]. Moreover, MACC1 has also been established as a negative prognostic marker in genitourinary (kidney, bladder, ovarian, cervical and endometrial cancer), skin, breast, lung, head and neck cancer (head and neck, tongue, nasopharynx, salivary gland) as well as glioblastoma and retinoblastoma [17–29]. Glioblastoma patients with low MACC1 expression receiving standard therapy survived longer compared to high MACC1 expressing patients receiving the same therapy (MACC1 low: 22.6 months vs MACC1 high: 8.1 months) [18]. In epithelial ovarian cancer, it was shown that high MACC1 expression led to reduced OS and progression-free survival in patients. In this study, tissue MACC1 mRNA expression was 2.5 times higher compared to normal surface ovarian epithelium [22]. Furthermore, a study on endometrial carcinoma found that serum levels of MACC1 and c-Myc were significantly higher in the experimental group compared to the control group and were associated with primary infiltration grade and LNM or distant metastasis [25]. Recently, the prognostic value of MACC1 was demonstrated in cutaneous melanoma, showing higher levels of MACC1 expression in metastatic melanoma compared to primary melanoma. However, no significant connection was found for other clinicopathological features such as histological subtypes, depth of invasion and staging [28]. Furthermore, higher MACC1 expression levels were associated with differentiation and blood vessel invasion in patients with small invasive lung adenocarcinoma, leading to a significant reduction in OS and disease-free survival [29]. In addition to the direct investigations performed in individual cancer entities, comprehensive bioinformatic pan-cancer analyses further confirmed these findings [27, 30]. Hu et al. found that abnormal DNA methylation might be a major cause for the different expression of MACC1 over various cancer types. Furthermore, a possible relation between MACC1 and T-cell function and the polarization of tumor-associated macrophages (TAMs) was hypothesized, indicating a potential immune therapeutic target for many different malignancies [27]. Of particular interest is the role of MACC1 in early-stage cancers. These data indicate that MACC1 has the potential to identify tumors with high metastatic potential even at an early stage, before spread of the primary tumor to distant organs is clinically detectable. This is of special relevance in situations, where the identification of high-risk patients might lead to an earlier escalation of therapy and potentially increase patient survival [15, 31].

Of note, MACC1 is not only detectable in tumor tissue; but can also be detected in blood samples, serving as liquid biopsies. A number of studies have demonstrated the feasibility of detecting MACC1 transcripts in the blood of patients and have shown a significant association with poor survival, confirming data generated in tumor biopsies [18, 32–36]. Importantly, the ability to detect MACC1 levels in blood samples opens up new possibilities in diagnostic procedures. In a study by Ahmed et al., MACC1 levels in the blood were used to differentiate between benign and malignant breast disease with a sensitivity of 96.7% and a specificity of 92.5% [37]. Another study demonstrated that patients with colorectal adenomas could be distinguished from healthy subjects based on analysis of blood MACC1 levels (sensitivity = 67%, specificity = 71%) [38].

In addition, there is growing evidence that MACC1 may also be a predictive marker of response to chemotherapy. In vitro analyses have demonstrated that MACC1-expressing colon cancer cell lines exhibit poor response to irinotecan, 5-fluorouracil (5FU), and cisplatin [39, 40]. Furthermore, the expression of MACC1 in CRC cell lines was associated with a poor response to doxorubicin in in vitro and in vivo experiments [41]. This important finding was also confirmed in other cancer entities, with MACC1-high expressing gastric cancer (GC) cells demonstrating a poor response to oxaliplatin, whereas ovarian and lung cancer cell lines exhibited a poor response to cisplatin, and pancreatic cancer cells poorly responded towards gemcitabine treatment [42–45].

Lastly, MACC1 has been recently implicated in other, non-cancerous diseases. First suggestions in this regard arise from the findings of an association between MACC1, Schwannoma and deafness [46], the emerging context of MACC1 and cancer-associated depression (affecting the catecholamine pathway) [47] as well as a correlation of MACC1 and pulmonary arterial hypertension [48].

Taken together, this highlights MACC1 as an important clinically relevant driver of tumor progression and metastasis in a wide range of tumor types. The high impact of MACC1 expression levels on patient survival and the ease of detection in tumor tissue or blood samples make MACC1 an ideal biomarker for use in the clinic. Moreover, the preclinical analysis of the role of MACC1 for therapy resistance could be a valuable tool for further optimization of currently available therapy strategies. Since the major multidrug resistance gene ATP-binding cassette sub-family B member 1 (ABCB1) is a direct transcriptional MACC1 target, statin treatment (lowering MACC1 levels and thereby ABCB1 as well) restores chemosensitivity [41]. Such approaches fuel the hope to offer patients more effective treatment options in the future which may render the cancers more chemosensitive. A combination of MACC1 specific therapies with standard chemotherapy might increase the treatment outcome significantly and future research needs to show the feasibility of such combinatorial approaches. By continuing to develop specific MACC1-targeting therapies, effective anti-metastatic drugs will offer patients more effective treatment options in the future. Importantly, to efficiently target MACC1 and its biological functions, one crucial step is to understand its protein structure as well as its role in cellular processes and signaling pathways.

## Form and function: MACC1 structure determines its interactions

The structure of MACC1 is well characterized, consisting of 852 amino acids, with an N-terminal intrinsic disorder region followed by a ZU5, a SRC Homology 3 (SH3), and double-death domains close to the C-terminus [4, 5, 49, 50]. The ZU5 domain of MACC1 closely resembles ZU5 domains in other proteins such as Ankyrin (ANK) or the Netrin receptor (Unc5b) [51–54]. Interestingly, ZU5 domains are often found along with death domains. In contrast to most other ZU5 domain-containing proteins, which only possess one death domain, MACC1 exhibits two partially overlapping death domains [4, 5, 49, 50, 54].

Based on sequence similarity analysis, the closest homolog of MACC1 is SH3 domain-binding protein 4 (SH3BP4; alternatively TTP), a protein involved in cargo-specific regulation of CME [5, 55, 56]. A recent study by Imbastari et al. evaluated the association of MACC1 with CME, establishing that MACC1 also interacts with endocytic accessory and cargo proteins involved in various steps of CME. The authors showed that cells overexpressing MACC1 increase the rate of epidermal growth factor receptor (EGFR) recycling and impaired receptor degradation in a CME-dependent context [55]. Importantly, MACC1 directly interacted with clathrin heavy chain (CLTC), dynamin 2 (DNM2) and adaptor protein 2 (AP-2) and thereby allowed the rerouting of internalized receptors towards recycling, as shown exemplarily for the transferrin receptor (TfR) and the EGFR. In this context, particularly the SH3 domain was found to be crucial for this interaction of MACC1 with TfR and CME factors. Consistently, deletion of MACC1´s SH3 domain impaired TfR internalization in CRC cell models [55]. Substantiating the importance of the SH3 domain, previous studies have reported that deletion of the MACC1´s SH3 domain impacts MET expression—a central positive feedback loop mediated by MACC1— as well as metastasis formation in mouse models. Together, these studies underline the crucial role of the SH3 domain in facilitating the pro-metastatic functions of MACC1 [4, 5, 55].

Given the essential role of the SH3 domain in MACC1-mediated metastasis in conjunction with the confirmed presence of proline-rich motifs within the MACC1 sequence, it was hypothesized that the SH3 domain may be involved in intramolecular interactions leading to homomeric or heteromeric dimers [56]. Interestingly, a study by Tosoni et al. indicated that SH3BP4 exists as a dimer and the SH3BP4 dimers could act as a scaffold coordinator coupling TfR-coated pits with dynamin [57]. Several studies have demonstrated advantages associated with protein oligomerization such as diversity, specificity, and stability [58–60]. It is implicated that many proteins in biological systems exist as dimers or higher order oligomers and are responsible for regulating a myriad of biological processes ranging from enzyme to gene regulation [58, 61–64]. In this context, a recent study by Dumbani analyzed the ability of MACC1 to dimerize and its impact on MACC1-mediated metastasis phenotype. Using a combination of AlphaFold-Multimer [65–67] and BRET [68–71], the study evaluated the dimerization ability of MACC1. The current findings describe a ZU5-dependent formation of MACC1 homodimers in CRC and a human embryonic kidney (HEK)293 cell model system. The data revealed that specific residues present in the first β-sheet of the ZU5 domain play a crucial role in the formation of MACC1 dimers [72]. Specifically mutating these residues did not only hinder MACC1 dimerization but also impacted MACC1-mediated metastatic capacities. Further investigations addressing the regulation of MACC1 dimerization and different oligomeric states of MACC1 in tumor cells would be highly beneficial [72]. Focusing on modulating MACC1 dimerization—therein either destabilizing active signaling complexes or promoting MACC1 degradation—may open up novel avenues for specific anti-metastatic therapies which could impede MACC1-driven metastasis. Altogether, these recent findings point towards the presence of MACC1 homodimers in living cells and identified the residues important in facilitating MACC1 dimerization.

Conclusively, research in the past decade allowed for a better understanding of the key players involved in MACC1-mediated metastasis and provided new insights into the MACC1 structure. Further research exploiting the spatiotemporal regulation of MACC1 and the precise mechanism of MACC1 in regulating metastasis, but also physiological functions would help refine the MACC1 signaling landscape and develop more effective therapies particularly targeting metastasis.

## MACC1 – a master regulator of cancer-associated signaling pathways

**Fig. 2:**
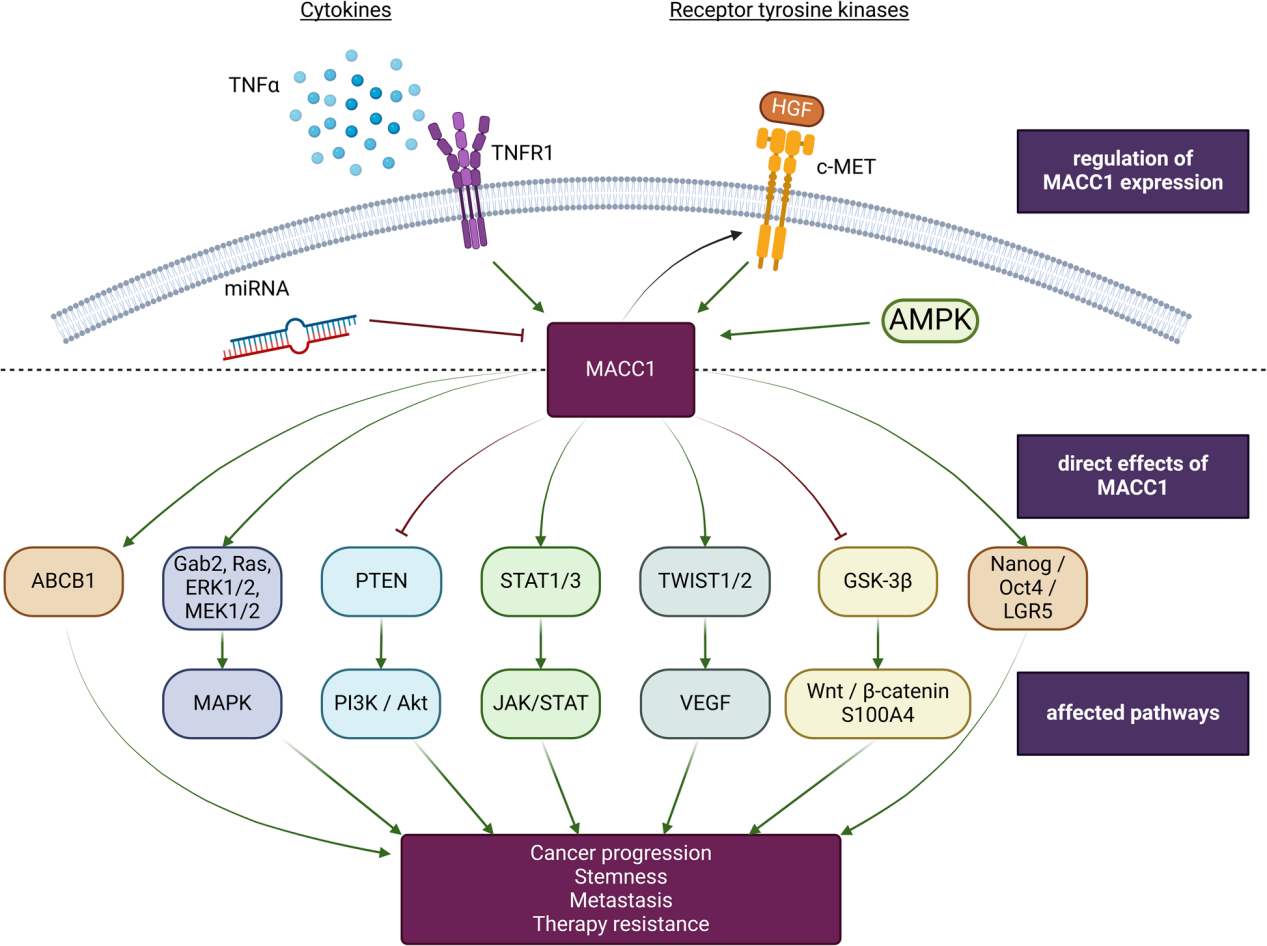
Regulation of MACC1 expression and its impact on signaling pathways. The expression and function of MACC1 is regulated by receptor tyrosine kinases such as HGF/c-MET and cytokine receptors including TNFR1, but also through micro-RNAs and the lncRNA MACC1-AS1, functioning via AMPK. Downstream targets of MACC1 include MEK/ERK, PI3K/Akt, STAT1/3, TWIST/VEGF, Wnt/β-catenin and the stemness genes NANOG/Oct4/LGR5. Created with BioRender

Overexpression of the MACC1 gene is highly predictive for malignant tumors with a high risk for metachronous metastases up to 12 years after removal of the primary tumor [4]. Generally, the expression of MACC1 has been detected in multiple different cancers, however it is not limited to aggressive tumors but can also be found in Schwannomas or benign colon adenomas. Moreover, the expression levels can greatly vary, ranging from almost complete absence to massive overexpression. Contrastingly, the expression of MACC1 is hardly detected in normal tissues and its physiological function remains largely unknown [5, 38, 56]. Within the tumor, MACC1 is predominantly expressed in the tip cells located at the invasive front and bordering the normal surrounding tissue. Furthermore, MACC1 expression was confirmed in tumor buds, i.e. cell clusters that have scattered further into the adjacent stroma [73]. Importantly, a previous report from Zincke et al. suggested the presence of MACC1 signalosomes, able to recruit and activate downstream effectors of receptor tyrosine kinases (RTK) as one key feature of MACC1´s malignant function. This study revealed a MACC1-mediated increased activation of several key players involved in cell motility and cell survival and proposed an important role of SRC in MACC1-dependent tumor progression [49, 74]. Moreover, the expression of MACC1 and its functions are deeply entangled with pathways of membrane-bound receptors for growth, inflammation and cell death [34, 73] (Fig. 2). Specifically, MACC1 is closely tied with the following signaling pathways:

### MACC1 as transcriptional regulator

The MACC1 protein—which features protein domains seen in other known signaling factors such as SH3BP4, ANK, UNC5 and UNC5CL—is able to translocate into the nucleus. Despite lacking confirmed DNA-binding protein domains [50], MACC1 attaches to the promoters of several target genes, inducing e.g. the MET gene, which encodes the receptor tyrosine kinase c-MET [4, 75], or the promoter of S100P [76], a calcium-binding member of the S100 protein family. Similarly, the transcription factor specificity protein-1 (SP1) binds to promoters of MET and S100P and is essential for S100P regulation by thioredoxin (TXN) [77–80]. Interestingly, MACC1 binds closely to SP1 binding sites on the MET and S100P promoters [75, 76] and TXN has recently been found to interact with MACC1 [55]. This points towards a connection or even a regulation of MACC1 with the confirmed transcription factor SP1. However, it remains to be resolved whether MACC1 can directly affect the presence of transcription factors on binding their target sequences.

### MACC1, HGF/c-MET and many more

The capacity of MACC1 to upregulate c-MET expression transcriptionally has been shown in CRC, ovarian cancer (OC), pancreatic cancer (PDAC) and osteosarcoma [4, 81–83]. Interestingly, MACC1 was able to induce c-MET expression in the U-2OS sarcoma cell line but not in non-cancerous human umbilical vein endothelial cell (HUVEC), indicating that MACC1 requires the presence of c-MET-specific transcription factors [81]. Of note, MACC1 overexpression in carcinoma-associated fibroblasts (CAF) was found to promote the secretion of chemokines and the c-MET ligand HGF, stimulating lung cancer cell invasion via the paracrine activation of c-MET [84]. Functionally, the key feature of the HGF receptor c-MET is the facilitation of scattering, proliferation, and branching morphogenesis in development, tissue regeneration and cancer. Furthermore, c-MET induces other signaling cascades such as Rat sarcoma/mitogen-activated protein kinase/extracellular signal-regulated kinase (RAS-MAPK-ERK), phosphatidylinositol 3/kinase-protein kinase-B (PI3K-Akt/PKB), and—secondary via the mentioned pathways as well as directly—the Wingless/integrin (Wnt)/β-catenin pathway [85–87].

### Participation of MACC1 in the Wnt/β-catenin pathway

Wnt/β-catenin signaling is a well-studied signaling cascade with interaction points including c-MET or other receptor tyrosine kinases (e.g. EGFR) as well as PI3K/Akt in response to inflammatory factors [88]. In physiological states, the mediator β-catenin is prevented from nuclear translocation by continuous proteasomal degradation regulated by the so-called destruction complex. The canonical Wnt pathway describes binding of extracellular Wnt ligands to Frizzled (Fz) and lipoprotein receptor-related protein (LRP). Both proteins belong to the destruction complex together with Axin, adenomatous polyposis coli (APC), casein kinase 1 (CK1) and glycogen synthase kinase 3 beta (GSK-3 beta). Upon induction of the pathway, the destruction of β-catenin is hindered allowing it to move into the nucleus [89]. Upon nuclear translocation, β-catenin can induce the Wnt target genes and thereby promote cellular proliferation, migration, invasion and stemness.

The dysregulation of the Wnt/β-catenin pathways by MACC1 has been confirmed in multiple cancer entities. In nasopharyngeal cancer, MACC1 overexpression was followed by the upregulation of Wnt targets such as c-MET as well as an increased phosphorylation of Akt, whereas ERK1/2 remained unaffected [90]. In CRC, MACC1 induces an inhibiting phosphorylation of GSK-3 beta, which permitted an increased nuclear translocation of β-catenin, leading to a downregulation of E-cadherin and an upregulation of the Wnt targets cyclin-D1 and c-Myc [91]. Additionally, MACC1 mediates the phosphorylation of β-catenin at Ser552, which intensifies its interaction with T cell factor 4 (TCF4) and upregulates cyclin-D1, matrix metalloproteinases (MMP)2/9 and S100A4 [92]. Intriguingly, MACC1 can also directly induce several β-catenin target genes. The APC^Min^ mouse model for colon cancer displays a Wnt-driven development of multiple colonic adenomas. Importantly, APC^Min^ tumors of mice with additional MACC1 overexpression in the intestinal mucosa (villin-MACC1/APC^Min^) transformed these adenomas into invasive adenocarcinomas. Comparing vil-MACC1/APC^Min^ and APC^Min^ tumors of these mice by transcriptomics, it was found that MACC1 directly binds to the promoter of NANOG, a target of activated Wnt signaling functioning as a stemness factor [93] and furthermore also an upregulated expression of PLAU and octamer-binding transcription factor 4 (Oct4) [7]. In CRC, it was recently shown that MACC1 also upregulates ALDH1 [94, 95] and Leucine-rich repeat-containing G-protein coupled receptor 5 (LGR5) by binding to the regulating promoter regions [96, 97]. While these findings are intriguing, they are also puzzling as they suggest that MACC1 can circumvent the kinase pathways elaborated above. This was further corroborated in pancreatic cancer, where the c-MET inhibitor JNJ-38877605 did not prevent direct protein–protein interaction of MACC1 with the EMT-marker SNAI1, resulting in the upregulation of fibronectin 1 (FN1) and a repression of E-cadherin [11].

### MACC1 and the MAPK/ERK signaling

Upon HGF binding, c-MET employs the adapter proteins growth factor receptor-bound protein 2 (GRB2) and SRC homology 2 domain-containing (SHC) to activate Ras, leading to MAPK/ERK activation and subsequently an increase in cell scattering [86]. Importantly, MACC1-overexpression enabled CRC cells to undergo this scattering by directly promoting HGF/c-MET signaling. This effect was abrogated by the application of specific MEK-inhibitors UO126 and PD98059, while the PI3K/Akt inhibitors Wortmannin and LY294002 had no effect [4]. Furthermore, via promoting CME, the overexpression of MACC1 triggered EGFR activation and internalization. Subsequently, this lead to a pronounced increase of EGFR phosphorylation (Y1068) which—via enhanced interaction with GRB2—upregulated MEK/ERK signaling [55].

Interestingly, in pancreatic cancer MACC1 can also convey chemoresistance towards gemcitabine by stabilizing the expression of Gab2, Ras and pERK1/2 [45]. Moreover, in ovarian and cervical cancer, MACC1 increases the phosphorylation of MEK1/2 and ERK1/2, thereby increasing proliferation and suppressing cellular apoptosis [42, 98]. In GC, MACC1 was found to upregulate c-MET and the EMT factor TWIST1/2, which induced VE-cadherin and vascular Endothelial Growth Factor (VEGF) receptor 2 expression leading to vasculogenic mimicry as a key mechanism underlying tumor associated neovascularization [99, 100]. Whilst the immediate involvement of MEK/ERK was neither excluded nor confirmed, in breast cancer it has been reported that MEK/ERK signaling can directly regulate TWIST1/2 via c-Jun N-terminal kinases (JNK) in response to transforming growth factor (TGF)-β stimuli [101]. Importantly, the phosphorylation of MACC1 by MEK1 leads to its stabilization and thus, a self-enforcing MACC1/MEK/ERK signaling [49].

### MACC1 association with PI3K-Akt/PKB signaling

PI3K can either directly interact with c-MET or through the association with the adapter protein Gab1. Functionally, this relays HGF signals towards the Akt/PKB pathway. Engagement of this pathway in turn activates proliferative and migratory signal cascades downstream of mammalian target of rapamycin (mTOR) and nuclear factor kappa B (NF-κB) [86, 87]. Administering c-MET inhibitors prevented MACC1-driven cell migration in lung cancer and the induction of Akt and VEGF C/D by MACC1 in GC [102–104]. Moreover, studies in hepatocellular cancer (HCC) confirmed that a MACC1-dependent increase of phosphorylated Akt was indeed sensitive to PI3K-inhibition [105]. Within this pathway, phosphatase and tensin homolog (PTEN) dephosphorylates PI3K and is an important physiological adversary of PI3K/Akt signaling [106]. Suppression of PTEN expression by MACC1 was observed in esophageal cancer (EC) and in HCC. Importantly, MACC1-overexpression was shown to increase secretion of IGFBP2, which mediates an integrin-dependent repression of PTEN activity [107–109]. Aside from upregulating c-MET kinase or suppressing PTEN, MACC1 seems to directly interact with YWHAE (14–3-3 epsilon), a newly identified activator of PI3K/Akt signaling [110, 111].

Furthermore, high glucose abundance is capable of inducing MACC1 overexpression in CRC, which in turn stimulates the translocation of glucose transporter (GLUT)1 from endosomal pools through PI3K/Akt1 and thus functionally enhances glycolysis by promoting the so-called Warburg effect [112]. In GC however, adenosine monophosphate kinase (AMPK)-dependent MACC1 overexpression is observed after glucose deprivation as well as exposure to acetylcholine (the paracrine neurotransmitter of the vagal nerve). Of note, the upregulation of MACC1 expression can also be detected in trastuzumab-resistant cancer cells, where it induced the core enzymes of glycolysis hexokinase‑2 (HK2) and lactate dehydrogenase A (LDHA) through PI3K/Akt [113–115].

### NF-κB, inflammation and MACC1

The induction of an inflammatory processes is a hallmark of cancer and elicits canonical NF-κB signaling in most tissues [116, 117]. In CRC associated with inflammatory bowel diseases (IBD), stimulation of the tumor necrosis factor (TNF) receptor 1 by TNF-α led to a NF-κB activation and c-Jun-dependent transcriptional upregulation of MACC1. A process that was reversible by the addition of the TNF-α blocking antibody adalimumab [118]. In fact, the Activator protein 1 (AP-1)/c-Jun-mediated MACC1 regulation inspired a continuous quest for the development of specific inhibitors of MACC1, which has identified the very commonly used statin drugs as well as identification of novel MACC1 inhibitors to restrict metastasis formation [119, 120].

Furthermore, the NF-κB pathway mediates HGF/c-MET-dependent proliferation and tubule formation, and involves the phosphorylation of inhibitor of κB kinase (IKK) and the activation of its effector p65 [121, 122]. These processes were observed in cervical cancer downstream of MACC1, leading to induction of MMP2/9, functionally relevant for the degradation of the extracellular matrix and well-studied players in cancer metastasis [123].

### JAK-STAT signaling, anti-apoptosis and MACC1

To facilitate anti-tumor immunity, tumor reactive T-cells will induce cellular apoptosis in tumor cells via an engagement of the death receptor Fas and its ligand. Importantly, cell-death resistant cancer cells can repress the expression of this receptor through modulation of the Janus kinase (JAK)/signal transducer and activator of transcription (STAT) signaling pathway [116]. In this context, MACC1 increases the activation of STAT1 and STAT3 as well as Mcl-1, a target gene of the JAK/STAT signaling pathway which mediates anti-apoptotic effects including the repression of Fas transcription. Exposure to the JAK-inhibitor ruxolitinib can prevent the induction of Mcl-1 and thereby restores Fas-ligand-dependent apoptosis in CRC cells [124].

### Interplay of MACC1 and mTOR

In eukaryotic cells, the kinase mTOR can communicate the arrival of new exogenous nutrients, induce anabolic metabolism and inhibit autophagic as well as proapoptotic processes [125]. In pathologically deregulated signaling states, mTOR activation can be inadequately regulated, propagating immune evasion and cell growth [126]. Of note, by systematically applying small molecule inhibitors targeting c-Met, Akt and mTOR in GC cells, a connection between MACC1 and c-MET/Akt/mTOR signaling was identified, in which MACC1 also mediated the upregulation of programmed cell-death ligand 1 (PD-L1) [127]. In glioblastoma stem cells the induction of autophagy limited tumor growth in vitro and in vivo, with a knockdown of MACC1 suppressing the phosphorylation of mTOR and further inducing the autophagy-associated genes LC3-II and beclin [128].

## MACC1, cancer stemness and metastasis

The ability of cancer stem cells (CSC) to promote tumor initiation, progression, relapse, invasiveness and metastasis as well as chemo- and radioresistance have been repeatedly reported in the last decades [129]. CSCs are a small population of all cancer cells identified by using biomarkers such as cluster of differentiation (CD)44, CD166, NANOG, Oct4, ALDH1, and LGR5, and can effectively promote cancer onset, spread, and therapy resistance. Of note, these biomarkers can potentially be targeted to attack cancer at this important CSC stage. Remarkably, these features—cancer initiation, progression, invasiveness, metastasis as well as resistance—are phenotypes, which can be caused by MACC1 in different cancer entities. Thus, stemness and MACC1 are likely connected and especially understanding the impact of MACC1 on cancer stemness properties is of the highest interest.

In CRC, the association of MACC1 and stemness was reported, demonstrating i.e. the involvement of Forkhead Box A3 (FoxA3), a DNA-binding transcription factor [130], and micro RNA (miR)−3163, which has MACC1 as its direct target [131]. Moreover, the interaction of MACC1 with the CSC marker Doublecortin-like kinase protein 1 (DCLK1) kinase, which possesses the capacity to phosphorylate MACC1, contributes to metastasis formation in various tumor entities [132]. MACC1 also facilitates CSC-like properties in CRC cells through PI3K/Akt signaling [133]. Furthermore, in CRC cells DBC1 increases Wnt/β-catenin mediated MACC1 expression in colonospheres, thereby promoting sphere-forming abilities [134]. Together with CD44, Twist1 and Kiss-1, MACC1 was found to be of prognostic value for CRC metastasis [8].

In a recent study, another novel link of MAC1 and cancer stemness was discovered by using 2D as well as 3D cultures of human CRC cell lines, CRC-PDX mouse models and human CRC patient samples. This study revealed the regulation of LGR5 expression, an important biomarker for the identification of stem cells in the small intestine and colon, by MACC1 [96, 97, 135, 136]. Furthermore, higher concomitant expression of MACC1 and stemness genes was observed in CD44^high^ and ALDH1^+^ stem cell populations. Here, direct binding of MACC1 as a transcription factor to the LGR5 gene promoter was confirmed using forced MACC1 expression, knockdown or knockout of MACC1. Importantly, these changes in MACC1 expression impacted the expression of stemness as well as phenotypic features such as the initiation of tumor sphere formation and clonogenicity. This newly discovered context was confirmed by MACC1-LGR5 expression correlation analyses in four independent CRC patient cohorts. Taken together, this proves that MACC1 promotes CSC-like properties as a novel signaling mediator in CRC via the employment of LGR5.

The causal link of LGR5 and cancer metastasis was furthermore shown by de Sousa e Melo et al. [137]. In this study, organoids were orthotopically injected into the colon mucosa of mice, leading to tumors which disseminated primarily to the liver. Here, LGR5-GFP^+^ cells were enriched in micro-metastasis compared to primary tumors and macro-metastasis, supporting the notion that dissemination and/or colonization at distant sites may originate from LGR5^+^ CSCs. The MACC1-LGR5 link is also described by Merlos-Suarez et al. [136], reporting that the intestinal stem cell signature—using e.g. LGR5 to identify CSC—can predict disease relapses. Interestingly, in this study the tumor-promoting and metastasis-inducing MACC1 is upregulated in LGR5^+^ stem cells of the mouse intestine [136]. However, in the more recent study by Erdem et al., stem cell populations were enriched by CD44 or ALDH1 sorting. This approach could not only demonstrate a simple association of MACC1 and LGR5 expression, but more importantly the molecular mechanism of MACC1 binding to the LGR5 gene promoter. This binding in turn initiates the expression of LGR5 and therein induces stem cell properties, such as tumor sphere formation [96].

In recent years, MACC1 gene expression has been investigated in the context of stemness in various solid cancer entities. For instance, MACC1 has been found, together with ALDH1, as a prognostic biomarker for non-small cell lung- [94], hepatocellular- [138] and also ovarian cancer [139]. In lung cancer, the long non-coding (lnc)RNA MACC1 antisense RNA 1 (AS1) was reported to enhance stemness properties by promoting UPF1-mediated destabilization of LATS1/2 [140]. The same lncRNA MACC1-AS1 promoted stemness by antagonizing miR-145 in hepatocellular carcinoma cells [138], and via suppressing miR-145-mediated inhibition on SMAD2/MACC1-AS1 axis in nasopharyngeal carcinoma [141]. In cervical cancer, MACC1 has been described to induce not only migration and invasion via the Akt/STAT3 pathway, but also stemness as illustrated by sphere formation assays and the expression of stemness factors such as NANOG and Oct4 [142]. In GC, the regulation of MACC1 promotes stemness and chemoresistance through elevated fatty acid oxidation [143]. In sebaceous gland carcinoma, the expression of CD133 and MACC1 was positively associated with local invasion, LNM, and TNM stages [144]. Interestingly, in head and neck cancer the expression of MACC1 was found to be higher in CSC when compared to cancer cells. This suggests that it accelerates the development of CSC from cancer cells in patients with malignant tongue cancer (belonging to head and neck cancer types) [145]. In addition, CD133^low^ retinoblastoma (Y79) stem cells showed a higher expression of several embryonic stem cell genes (HOXB2, HOXA9, SALL1, NANOG, Oct4, LEFTY), stem cells/progenitor genes (MSI2, BMI1, PROX1, ABCB1, ABCB5, ABCG2), and the metastasis related gene MACC1, when compared to CD133^high^ cells [146].

In this context, the link of MACC1, chemoresistance and of stemness is important. Several reports are referring to chemotherapeutic drugs affecting the relation between MACC1 expression and stemness. For instance, FoxA3 was described as a potent tumor suppressor in CRC, which may disrupt the maintenance of stemness and modulate the sensitivity to oxaliplatin by inhibiting the transcription of MACC1 [130]. Moreover, restored chemosensitivity for 5-FU was investigated in MACC1-depleted CRC cells, leading to reduced sphere formation and expression levels of pluripotent markers, including CD44, CD133 and NANOG [133].

Taken together, the discovered association of MACC1 with stemness genes leads to a more comprehensive understanding of the stemness-tumor progression/metastasis context. Thus, MACC1 is not only an inducer of metastasis and acts as a CSC-associated marker but is also a regulator of stem cell properties. Since CSCs are believed to be responsible for tumor metastasis and relapse, interventional approaches targeting MACC1 [119, 147], will potentially also target additional stemness genes. Consequently, this will further improve targeted therapies for cancer patients with the aim to eradicate CSCs, fight chemoresistance, prevent cancer recurrence and distant metastasis formation.

## MACC1 manipulates anti-cancer immunity and mediates immune evasion

Although the role of MACC1 in facilitating metastasis has been well documented since its discovery and within the last five years, links of MACC1 with tumor immune evasion strategies are still not very well defined. However, furthering the understanding of MACC1-related influences on immunological processes in cancer is essential as the successful avoidance of immune destruction is a key prerequisite for cancer cell spread within the body. To date, only very few studies report a relation between MACC1 and immunological processes. Most notably, one paper identified a close relationship between MACC1 expression levels and the immune checkpoint molecule PD-L1. This study analyzed GC tissues and cell lines, in which a MACC1 up- or downregulation led to mirrored changes in PD-L1 levels. This then perpetuated in a co-culture setting together with peripheral blood mononuclear cells (PBMCs), for the first time describing an immune-protective effect for cancer cells initiated by MACC1 [127]. Additionally, there are two studies reporting that MACC1 influences the intra-tumoral infiltration of immune cells. In colon adenocarcinoma (COAD), using the cancer genome atlas (TCGA), a positive correlation between MACC1 mRNA and the intratumoral levels of natural killer (NK) cells, neutrophils, and macrophages, but not with T cells (incl. CD8^+^, CD4^+^ and regulatory T cell subpopulations), B cells, and dendritic cells was described. The same study used the gene expression profiling interactive analysis (GEPIA2) database and reported that MACC1 mRNA correlated with biomarkers of immune cells, specifically NK cells, M1 and M2 macrophages, neutrophils and dendritic cells [148]. A second study performed in breast cancer reported somewhat conflicting results. Here, mRNA levels of MACC1 were positively correlated with TAMs but showed a negative connection with NK cell and CD8^+^ cytotoxic T cell infiltration [17]. To date, these findings allow a first, but limited assessment of how MACC1 influences immune evasion strategies of cancer cells. More experimental and clinical studies are needed to further elucidate the connection between metastatic potential of cancers and their immune evasive capacities. However, there are additional factors manipulated directly by MACC1 that then on their own can alter immune cell infiltration, differentiation, and overall anti-tumor immunity. These factors include a positive feedback loop of MACC1 towards c-MET, the stimulation of VEGF secretion, desensitization towards induced cell-death, induction of cancer cell stemness, alteration of cellular energetics, and the stimulation of multiple signaling cascades such as STAT, Wnt/β-catenin, and PI3K/AKT [149]. Alteration of these factors collaborate to mediate three main outcomes of MACC1-induced protection from immune cell destruction:

### Alteration of immune cell infiltration / polarization

One key mechanism by which MACC1 can affect the immunological recognition of tumor cells is through a regulation of cellular infiltration into the tumor mass as well as by affecting the polarization of specific immune cell subsets. Therein, HGF/c-MET can alter the infiltration of B and T lymphocytes, as well as promote anti-inflammatory cell subsets, including regulatory T helper cells (Treg) and M2 macrophages [150, 151]. Furthermore, cellular infiltration can be altered by a MACC1-mediated promotion of the Wnt/β-catenin pathway, which directly affects TAMs and reduces the recruitment of T cells into the tumor [152, 153]. Additionally, activation of the PI3K/Akt pathway has been shown to shift the cellular infiltration from cytotoxic T lymphocytes (CTLs) towards Tregs, M2 macrophages and myeloid-derived suppressor cells (MDSCs) [154, 155]. Lastly, MACC1 promotes the secretion of VEGF, which in turn can specifically promote the recruitment of Tregs and MDSCs into the tumor, simultaneously inhibiting the infiltration and cytotoxicity of CTLs and NK cells. This shift is—at least in part—mediated by alterations of blood-vessels, as VEGF decreases the expression of selectins as well as intercellular adhesion molecules (ICAMs) and vascular cell adhesion proteins (VCAMs), all collaborating to mediate immune cell extravasation from the blood stream into the tumor mass. Moreover, VEGF promotes the expression of CLEVER-1, which correlates with Treg and M2 macrophage infiltration, and Fas-Ligand (FasL) specifically decreasing the number of tumor-infiltrating CTLs, without inducing Fas-mediated apoptosis in Tregs [156–158].

### Promotion of an immuno-suppressive tumor microenvironment (TME)

Next to its impact on immune cell infiltration and polarization, MACC1—directly or via secondary factors—can induce an immune-suppressive TME. In this context, HGF/c-MET can alter the cytokine secretion towards an anti-inflammatory and immune-suppressive profile, reducing Interferon (IFN)-γ secretion and promoting the release of Interleukin (IL)−4 and IL-10 [150, 159]. Also activated PI3K/Akt signaling can lead to a shift in the cytokine milieu, specifically a reduction in pro-inflammatory cytokines IL-6 and TNF-α and the increased release of chemokine (C-X-C motif) ligand (CXCL)−17, which can induce the recruitment of anti-inflammatory cell subsets [155]. Furthermore, Wnt/β-catenin can affect the secretion of cytokines such as chemokine (C–C motif) ligand (CCL)4 and CCL5, reducing dendritic cell recruitment, while the secretion of Wnt-induced secreted protein 1 (WISP-1) promotes TAM and a pro-tumor TME [152, 160]. Moreover, MACC1 can modulate the TME through the induction of cancer cell stemness, as CSCs can secrete a multitude of immunosuppressive factors, including cytokines IL-4, IL-8, TGF-β, macrophage colony-stimulating factor (M-CSF), granulocyte–macrophage colony-stimulating factor (GM-CSF) but also extracellular vesicles (EVs) with respective cargos [161, 162]. These factors can then suppress anti-tumor functions and shift cellular polarization to induce a TME with favorable attributes for continuous cancer growth. Lastly, MACC1 regulates metabolic pathways and can promote autophagy, both contributing to an immune-suppressive TME by altering cytokine secretion and—through nutrient depletion, the release of toxic metabolites and increased acidity of the TME—excluding immune cells from the tumor mass [163–165].

### MACC1 impacts anti-tumor immune cell function

Altering the immune cell infiltration and polarization as well as creating an immune-suppressive TME collaborate with direct mechanisms of cellular interaction in reducing tumor-directed immune cell functions. The most prominent of those direct mechanisms is the expression of molecules termed immunological checkpoints. These cell surface proteins, forming a pair of receptor and ligand on both the tumor as well as the immune cell, directly inhibit immune cell effector functions. One of the key examples of this class of molecules is the pair of programmed cell death protein 1 (PD-1) and PD-L1. The expression of PD-L1 on cancer cells can be directly upregulated by MACC1 itself [127], or through secondary factors such as HGF/c-MET [166], via activated STAT1/3 [167], PI3K/Akt [155], or Wnt/β-catenin signaling [160], from metabolic alterations [168] or by promoting cellular stemness via Oct4/NANOG/LGR5 [169]. Additionally, Wnt/β-catenin [153] and cellular stemness [162] can also induce the expression of CD47, a negative regulator of macrophage activation and phagocytosis, therein acting as a checkpoint for innate immunity. Furthermore, the MACC1-mediated induction of STAT1/3 can downregulate the expression of Fas, a receptor involved in immune-cell induced apoptosis [124]. Another key mechanism to avoid immune cell recognition and destruction is the downregulation of tumor-surface antigens as well as major histocompatibility complex (MHC) molecules, which could present tumor antigens towards immune cells. In this context, CSCs have been shown to possess lower surface levels of MHC I [169] and it has been demonstrated that MACC1 can induce factors that regulate cancer stemness. Additionally, the expression of MHC I can be reduced through metabolic changes, specifically the deprivation of oxygen and available glucose [168], which can be affected by MACC1 expression, or via the MACC1-mediated promotion of endocytosis, a process which can degrade MHC I molecules [170]. Lastly, one key effector function of immune cells to destroy tumor cells is the release of granzyme B (GranB), a factor produced by CTLs and NK cells which induces apoptosis in target cells. In this context, MACC1 is able to reduce the susceptibility of tumor cells towards GranB by promoting autophagy and thereby effectively degrading this effector protein [165].

**Fig. 3:**
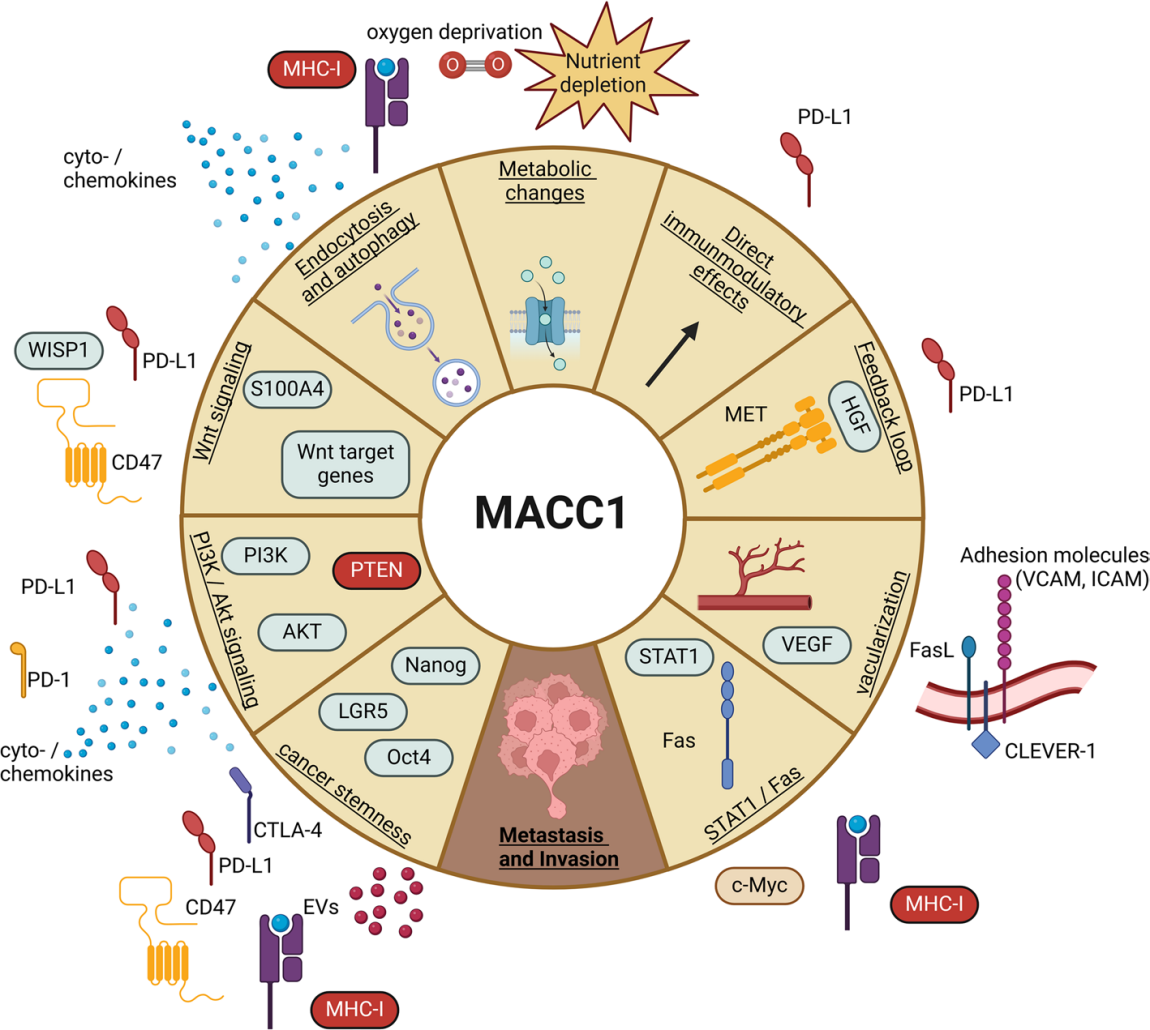
Direct and indirect effects of immunological consequence resulting from MACC1 expression. MACC1 is a regulator of many pathways possessing immunological outcomes. Direct effects of MACC1 expression are shown in the inner circle, whereas indirect consequences are aligned outside their corresponding slice. Negative effects initiated or mediated by MACC1 expression are indicated by red boxes. Created with BioRender

Taken together, there are several novel direct and indirect links of how MACC1 can mediate cancer immune evasion strategies (Fig. 3). Ultimately, additional extensive research is needed to further understand these processes and to connect MACC1 mediated metastasis formation with immune-related mechanisms.

## The contribution of MACC1 to therapy resistance

A large number of studies have elucidated that drug resistance is one of the main causes of treatment failure [171]. Although MACC1-related studies are mainly centered around the effect of MACC1 on tumor initiation and progression, several studies showed MACC1's impact on the outcome of cancer treatment [41, 115, 172]. In this context, elevated levels of MACC1 are closely linked to the efficacy of treatments in various tumor entities, including colorectal, gastric, and breast cancer [41, 115, 173].

Cytotoxic chemotherapeutic drugs are to this day the standard-of-care therapeutics for cancer. The majority of cancer patients are treated with chemotherapeutics during the course of their disease [174]. Various research groups have elucidated the effects of MACC1 on mechanisms of resistance towards chemotherapeutic drugs across a wide range of tumor types, including glioblastoma, colorectal, gastric, and pancreatic cancers [41, 115, 173, 175]. One key link between MACC1 and chemoresistance was first described mechanistically by Dahlmann et al. [41]. Here, the authors report the MACC1-mediated therapy resistance of CRC cells towards standard-of-care drugs via an upregulation of the ABCB1 protein, a key player in multidrug resistance (MDR). This upregulation inhibited the accumulation of chemotherapeutic agents in cancer cells, leading to therapeutic resistance against commonly used chemotherapeutics such as doxorubicin. Conversely, inhibiting MACC1 expression also decreased the expression of ABCB1, leading to elevated intracellular drug accumulation. Ultimately, this reduced treatment resistance and promoted cell death in malignant cells [41]. Of note, the clinical relevance of circulating cell-free ABCB1 transcripts (cfABCB1^tx^) were recently explored in plasma samples of ovarian cancer patients [176]. In this longitudinal study, cfABCB1tx levels were quantified in a total of 173 serum samples from 79 ovarian cancer patients using ddPCR. Increased cfABCB1^tx^ levels predicted poor PFS and OS with a strong correlation between cfABCB1^tx^ and the cell-free transcripts of its transcriptional activator MACC1 (cfMACC1^tx^). Combined assessment of cfABCB1^tx^ and cfMACC1^tx^ resulted in a further improved prognostic stratification at primary diagnosis, with the cfABCB1^tx^-high/cfMACC1^tx^-high phenotype being associated with the worst prognosis. Therein, this study reported for the first time, that circulating transcripts of ABCB1 are traceable in liquid biopsies, advancing a new dimension for systemic monitoring of ABCB1 expression in cancer patients.

Further studies have revealed that MACC1 also contributes to irinotecan resistance in CRC patients. In this context, the downregulation of MACC1 renders the cells more susceptible to irinotecan treatment [177]. However, the impact of MACC1 is not limited to CRC treatment. Subsequent studies have underscored the crucial role of MACC1 expression in cisplatin resistance in tongue squamous cell, ovarian and lung cancer through the activation of PI3K/AKT and ERK 1/2 signaling pathways [42, 104, 178]. The downregulation of MACC1 also decreased phosphorylated (p)ERK1/2 and BCL2 protein levels and upregulated the expression levels of BAD and BAX, making the ovarian cancer cells sensitive towards cisplatin treatment [42]. Furthermore, MACC1 induces the Warburg effect, which can promote the resistance towards trastuzumab, a monoclonal antibody commonly combined with chemotherapeutic agents as a first-line treatment in GC [115]. Another agent—gemcitabine—has been mainly used as a first-line therapy for pancreatic cancer patients [179]. Studies revealed that the lncRNA MACC1-AS1 regulates lipid oxidation and inhibits the ferroptosis induced by chemotherapeutic drugs such as gemcitabine [45]. Additionally, lncRNA MACC1-AS1 can mediate resistance mechanisms induced by Nuclear Receptor Binding SET Domain Protein 2 (NSD2) as displayed by Xue et al. in the context of esophageal SCC [172].

Another very common treatment option against cancer progression is radiotherapy (RT). More than half of cancer patients will receive RT at some point during the course of their disease. The aim of RT is to cause DNA damage and subsequently activate cellular death mechanisms in fast-dividing cells including malignant tumor cells. However, the majority of patients can develop resistance against RT approaches [180–182]. One crucial effect of MACC1 in treatment outcomes with RT is its role in DNA damage repair mechanisms. MACC1 promotes the repair of DNA damage caused e.g. by radiation, leading to cancer cell survival and progression despite the genotoxic stress induced by irradiation, as shown in a study with breast cancer patients. This capability of MACC1 contributes to the development of radioresistance in tumors, evading a substantial challenge to effective radiation therapy by involving to the lncRNA FGD5-AS1/miR-497-5p/MACC1 axis [180, 181].

Importantly, MACC1 is also involved in regulating immune checkpoints on tumor cells. This regulation significantly affects the response to therapies targeting these checkpoints, such as PD-1/PD-L1 inhibitors [149]. By influencing the expression of these checkpoints, MACC1 can alter the immunogenicity of cancer cells, impacting how effectively the immune system can recognize and target these cells [127, 149, 183]. This connection between MACC1 and the anti-tumor immune response opens up new aspects for research and possibly allows novel therapeutic strategies, especially for combining immunotherapies with MACC1-specific agents.

Another factor leading to drug resistance is initiated by the effect of MACC1 on cancer stemness. Many studies have shown the effect of the expression of cancer stemness genes on drug resistance. In this context, studies have elucidated the promoting effect of MACC1 on stemness properties via the induction of Oct4, NANOG and LGR5 [7, 96]. The contribution of cancer stemness factors, such as LGR5 to various chemotherapeutics, including 5-FU and doxorubicin via WNT signaling, has been demonstrated extensively [41, 96].

Taken together, comprehensive analysis reveals that MACC1 significantly influences treatment outcomes and efficacy. Its role in mediating therapy resistance establishes MACC1 as a pivotal biomarker for predicting drug responses. Consequently, targeting MACC1 may be integral to enhancing the success of cancer treatments.

## New avenues of metastasis restriction through MACC1 intervention

After the discovery of the MACC1 gene, it has been proven various times that the gene-specific targeting of MACC1 expression using small interfering RNA (siRNA), short hairpin RNA (shRNA) or micro RNA (miRNA) leads to reduced migratory properties and cellular invasion, while this simultaneously induces apoptosis, elevates chemo-sensitivity of cancer cells and ultimately also inhibits metastasis formation in vivo [5].

### The role of miRNAs in regulating MACC1 expression

In the last years, a large number of studies have worked towards untangling the miRNA network surrounding MACC1 and how miRNAs can impact MACC1 expression levels. miRNAs are short non-coding RNAs which are often highly dysregulated in cancers, with some being exclusive for distinct cancer entities. Increasing evidence from the last decades elucidated their oncogenic or tumor-suppressive functions [184]. In the context of MACC1-driven cancers, the miRNA network has been best studied in CRC and HCC. miR-338-3p has been identified to be downregulated in CRC cell lines and tissues, with the lowest expression levels in metastatic CRC cell lines such as SW620. Intriguingly, this cell line possesses a very high expression level of MACC1. Importantly, the OS of CRC patients was significantly shortened in miR-338-3p^low^ expressing groups. In a therapeutic context, miRNA-338-3p mimics were able to suppress cell proliferation, colony formation, migration, and induced apoptosis in CRC cell lines. Zou et al. showed a correlation in the expression levels between miRNA-338-3p and MACC1 [185]. Moreover, a miRNA-338-3p agomir reduced xenograft CRC tumor growth by MACC1 overexpressing HCT116 cells, a cell line of colorectal origin. Mechanistically, it was predicted that miRNA-338-3p can bind to the 3’ untranslated region (UTR) of MACC1. Using a luciferase assay, Lu et al. showed that MACC1 is a direct target of miRNA-338-3p leading to reduced cell proliferation, migration and G1/S cell cycle arrest [186]. In 2020, Zhang et al. could demonstrate with a dual-luciferase reporter assay that lnc-HSD17B11-1:1 may act as a competitive endogenous RNA (ceRNA) for miRNA-338-3p promoting MACC1-mediated proliferation and metastasis in CRC [187]. Additionally, miRNA-940 [188], miRNA-145-5p [189], miRNA-138-5p [190], miRNA-320a [191], miRNA-1236-3p [192], and miRNA-330-5p [193] have been shown to have a regulative impact on MACC1 expression levels in CRC. Moreover, it was demonstrated that targeting MACC1 via miR-940 enhanced the antitumor effect of anlotinib—a small-molecule multi-target tyrosine kinase inhibitor—effectively reducing proliferation and metastasis. Of note, using CRC cells and CRC xenograft mouse models, a strongly elevated effect on the reduction of tumor size and metastasis formation was seen in a combinatorial approach with miR-940 and anlotinib compared to single treatments [188].

In HCC, several different miRNAs have been characterized which can affect the expression levels of MACC1. In 2018 Cui et al. identified miR-18a-5p and miR-18b-5p to be upregulated in HCC tissue from patients compared to adjacent non-tumor tissue [194]. MACC1 was predicted in silico to be a possible target of both miRNAs. Moreover, the expression of miR-302 a/b/c was shown to be decreased in HCC tissue from patients and in HCC cell lines [195]. In this study, the authors could demonstrate through a luciferase reporter assay that miR-302 a/b/c can target MACC1 and suppress its expression levels. This then resulted in reduced migratory properties of HCC cell lines and decreased tube formation properties of HUVECs, effectively reducing the angiogenic potential of the cells. Further, miR-34a and miR-125a-5p have been explored by Zhang et al. in 2020 for their potential as a tumor suppressor in HCC [196]. Both miRNAs can target MACC1 directly and the overexpression of miR-34a and miR-125a-5p resulted in a downregulation of MACC1 expression. This translated into reduced activation of the PI3K/AKT/mTOR pathway leading to a decrease in proliferation and metastasis while inducing apoptosis in HCC cells. Next to the mentioned miRNAs, the lncRNA MACC1-AS1 was shown to promote stemness in HCC cells [138], nasopharyngeal carcinoma cells [141] and stemness and chemoresistance in GC cells [143]. In this context, antagonizing miR-145 and the lncRNA DDX11-AS1 [197] can accelerate the HCC progression via the miR-195-5p/MACC1 signaling by binding miR-195-5p which results in elevated MACC1 expression levels.

### lncRNAs affecting MACC1 expression levels

Recent research approaches on lncRNA MACC1-AS1 have demonstrated its significance in tumor progression in various cancer entities. Many lncRNAs have the ability to interact with miRNAs—as described above in the context of miR-145. Zhang et al. identified that lncRNA MACC1-AS1 is able to sponge multiple miRNAs such as miRNA-384 and miRNA-145-3p, both have been recognized for their tumor-suppressive actions. This sequestering lead to a change in the cell growth phenotype via an upregulation of PTN and c-Myc transcripts [198]. In breast cancer cells, a lncRNA-miRNA-mRNA regulatory network was identified, in which the interaction of lncRNA MACC1-AS1 with miRNAs affects the expression of their respective target mRNAs, therein dysregulating cancer cell growth [199]. Moreover, it was demonstrated in breast cancer cells that lncRNA MACC1-AS1 can form a complex with DEAD-Box Helicase 5 (DDx5), recruiting Specificity Protein 1 (SP-1) to the MACC1 core promoter and ultimately leading to an increase in MACC1 expression [200]. In cervical SCC the sponge effect has been shown for the miRNA-34a resulting in an upregulation of cyclin-dependent kinases (CDK) 6 which increased cell cycle progression and cell proliferation [201]. Moreover, it was shown that lncRNA MACC1-AS1 can regulate Paired-Box-Protein 8 (PAX8) in HCC cells promoting cell proliferation, EMT and invasion [202]. Additionally, its involvement in distant recurrence after surgical resection of HCC tumors via regulating TGF-ß1 expression was reported [203]. In the context of lung cancer, lncRNA MACC1-AS1 can increase cell proliferation [204] and promote stemness properties via the hippo pathway [140].

Several other lncRNAs and circular RNAs have been shown to play a role in the miRNA network targeting MACC1. The lnc-HSD17B11-1:1 [187], lncRNA FGD5-AS1 [205], circ-FOXM1 [206], circ_0006174 [190], circ_0101802 [192], circ_0001038 [21], LINC01703 [207], lncRNA-CYTOR [208] and lncRNA ZFAS1 [148] can act as ceRNAs for microRNAs targeting the expression of MACC1. However, more research is needed to untangle the complex network of miRNA, lncRNA and circular RNAs in the context of MACC1-mediated cancer. First clinical studies with miRNA mimics and antisense miRNA inhibitors are being conducted and the further research might elucidate the feasibility of this approach in the fight against cancers [209].

### Small molecule inhibitors (SMI) to therapeutically target MACC1

Aside from possible genetic interventions, several approaches have been made to establish MACC1-specific SMIs or inhibitors targeting pathways that induce MACC1 expression, with the ultimate goal to reduce metastasis formation. After identifying agents of the class of statins in 2017 as effective MACC1 transcriptional inhibitors [210] their potential to effectively block MACC1 gene expression and reduce functions mediated by MACC1—including cellular migration and metastasis formation in vivo—have been continued to be studied in cross-entity approaches. Mechanistically, statins reduce the expression of MACC1 by interfering with the DNA binding groove of the transcription factor AP-1. Lin et al. demonstrated that DCL3 can transcriptionally inhibit MACC1 expression in GC by affecting RhoA/JNK/AP-1 signaling, reducing glycolysis and survival under metabolic stress [211]. Treating GC cells with lovastatin decreased MACC1 gene expression and elevated DLC3 expression levels. The tumor growth and metastasis formation of intrasplenically xenografted MKN45 cells was reduced under lovastatin treatment in vivo. Conclusively, the authors proposed that lovastatin could serve as a promising therapeutic substance in GC attenuating the DLC3/MACC1 axis. In 2022, another high-throughput screen with a different pharmacological library using the MACC1 promoter coupled to a luciferase gene was conducted, identifying other statins including fluvastatin and atorvastatin as effective MACC1 transcriptional inhibitors. Functionally, these agents were capable of reducing MACC1-mediated cell proliferation and colony formation properties of CRC cells. Further in vivo experiments with intrasplenically transplanted HCT116 cells confirmed the efficacy of statins to reduce liver metastasis formation. Even more compelling was a retrospective, two-center, cohort-based study assessing the risk of cancer in statin-treated patients compared to matched control groups. In this dataset, several ten thousand electronic health records collected at the University of Virginia (UVA) and the Charité – Universitätsmedizin Berlin – were analyzed in a 1:1 matched study design. Here, the overall impact of statins on the prevalence of diverse cancer entities was assessed, with a statistically significant reduction of cancer development in statin-treated patients. This reduction in the cancer risk by about 2-fold might be attributed, at least in part, to a reduced MACC1 gene expression [147].

Moreover, lovastatin has been used by Thankamony et al. to target a more aggressive tumor cell population with high metastatic potential obtained from genetically heterogeneous triple negative breast cancer [212]. The higher metastatic potential was identified to be mediated by an increased MACC1 gene expression level. Furthermore, the authors demonstrated that lovastatin lowers MACC1 gene expression and diminishes cell proliferation in the highly metastatic tumor population.

Next to statins, inhibiting MACC1 targets or proteins upstream of MACC1 has been another strategy to interfere with functions mediated by MACC1. It was shown by Zhang et al. that MACC1 mediates chemo-resistance in lung cancer cells via the PI3K/AKT signaling pathway [104]. Treating lung cancer cells with the PI3K/AKT signaling inhibitor perifosine suppressed this pathway and lowered MACC1-mediated chemo-resistance. A combinatorial treatment with cisplatin and perifosine reduced the growth of lung cancer cells in vitro and in xenografted mouse models more effectively than single treatments, clearly showing the involvement of the MACC1-mediated activation of the PI3K/AKT signaling pathway on chemo-resistance. Another study in CRC cells analyzed the effect of verapamil on chemo-resistance towards the active metabolite of irinotecan (CPT-11) in the context of MACC1. Here, verapamil can downregulate MACC1 expression levels and this effect is likely the cause of an increased chemo-sensitivity displayed in cells and xenografted models using irinotecan combined with verapamil [177]. Furthermore, in 2020, the impact of MACC1 on the cellular biomechanics of glioblastoma was studied. This study identified that MACC1 overexpression increases the migratory speed, elasticity and reduces the contact area of glioblastoma cells. These effects were abrogated by applying the c-Met inhibitor crizotinib, demonstrating that targeting c-MET in MACC1 driven cancers might be of therapeutic value [213]. Moreover, Wong et al. studied the effect of parecoxib on MACC1 expression level and found that this anti-inflammatory compound can inhibit MACC1 gene expression. This is accompanied with an inhibition of c-MET expression and AKT phosphorylation in the CRC cell line DLD-1 likely playing an important part in the anti-migratory properties of parecoxib exhibited in the study [214]. Another study identified novel tetrazolo-pyridazine based small molecules from a high-throughput screen as effective MACC1 transcriptional inhibitors. These novel compounds inhibit MACC1 gene expression in a dose-dependent manner, leading to a reduction in migration and wound healing capabilities in CRC cells and reduced metastasis formation in mice. The inhibition of the NF-κB pathway was proposed as a possible mode-of-action of these compounds, however this novel approach needs further evaluation [119]. Recently, MEK1 emerged as a novel therapeutic target, acting as an upstream factor regulating MACC1-mediated functions. MEK1 can directly phosphorylate tyrosine residues of MACC1 which then in turn leads to an increased ERK1 activation. The MEK1 inhibitors AZD6244 and GSK1120212 reduced MACC1-induced metastasis formation in xenografted mouse models by decreasing the MACC1 tyrosine phosphorylation mediated by MEK1 [49]. The effect of MEK1 inhibition has also been shown in the context of esophageal and gastric adenocarcinomas with the MEK1 inhibitor selumetinib, effectively reducing the migratory properties of cancer cells and metastasis formation in MACC1^+^ xenografted mouse models [15]. Furthermore, a recent study of Sun et al. demonstrated that the GPX4 inhibitor RSL3, was able to downregulate MACC1 gene expression at the mRNA and protein level as well as reduce the expression of GPX4 in CRC cells [215]. Knockdown of MACC1 enhanced the ferroptosis induction of RSL3 which led to an increase of reactive oxygen species (ROS)-induced lipid peroxidation and reduced cell viability. Next to the great potential of SMIs to reduce MACC1 expression or function, several naturally occurring compounds have been shown to affect MACC1 gene expression.

### Further factors affecting MACC1 levels

In short, saffron and its component crocin were identified to restrict the proliferation and migration of CRC cells. It was shown that MACC1 interacts with the stem cell marker DCLK1, activating MACC1 by phosphorylation. Crocin was capable of downregulating DCLK1 leading to the restriction of MACC1 mediated functions [132]. Furthermore, curcumin—found in turmeric, a member of the ginger family—was recognized to reduce MACC1 expression in a concentration-dependent manner in CRC cell lines, resulting in a reduction of proliferation, migration, clonogenicity and wound healing [216]. Cantharidin—a substance of the terpenoid class secreted by many species of blister beetles—and its analogue norcantharidin, known from traditional Chinese medicine, were investigated for their effect on MACC1. Both agents were able to reduce MACC1 gene expression as well as functional outcomes of MACC1 expression such as migration and colony formation [217]. Conclusively, natural compounds possess the capacity to affecting MACC1 expression levels but additional research is needed to further understand the mechanism by which these compounds facilitate their inhibition on MACC1 and to evaluate their feasibility to supplement modern anti-cancer therapies.

Moreover, there are additional mechanisms capable of directly or indirectly lowering MACC1 expression levels. In 2022 it was observed that MACC1 expression is elevated in obese adults, positively correlating with greater body fat mass and body mass index. Dietary intervention and physical exercise were shown to reduce MACC1 expression levels. Moreover, in vivo studies using rats validated this finding as diet-induced obese rats had higher MACC1 levels compared to normal weight littermates, suggesting a diet-based intervention strategy as a possible additional approach to lower MACC1 expression in cancers next to pharmaceutical intervention, although this approach needs further evaluation [218]. In the same year it was found that in pancreatic cancer cells IFN-γ inhibited MACC1 expression in a time and concentration dependent manner, leading to reduced cellular proliferation, migration, and downregulation of phosphorylated AKT and phosphorylated mTOR. Overexpression of lncRNA MACC1-AS1 reversed these findings, implying that IFN-γ mediates its properties through a lncRNA MACC1-AS1/MACC1 axis involving AKT/mTOR signaling [219]. Surprisingly, treating CRC cell lines with the proliferation inhibitor mitomycin abolished the MACC1-associated effect on the collective migration speed, indicating that targeting proliferation in MACC1^high^ expressing tumors might allow additional effects on cell migration [220].

Taking all these new findings into account together with the vast functions mediated by MACC1 on metastasis formation of cancer cells, it is becoming clear that targeting MACC1 gene expression, activation or its functional consequences is of tremendous therapeutic value and might lead to an improved therapeutic benefit for cancer patients with a high risk for metastasis. Of note, as MACC1 is regulated in a circadian manner, the treatment time and regime might play an important role in creating an effective treatment regime [221]. Nonetheless, more studies are required to fully assess the role of circadian regulation and to devise the most effective therapeutic approaches against MACC1-driven cancers. Moreover, combinatorial studies with chemotherapeutic agents or targeted therapies might be of interest in the fight against MACC1.

### Combinational approaches of MACC1 intervention with established cancer therapies

Already in the 1940´s it became evident that single agents often show only transient effects. Especially the first generations of chemotherapeutic drugs often affected a broad spectrum of cells that mostly are actively in the process of cellular division. Despite their indiscriminate toxicity, these drugs remain the cornerstone of modern anti-neoplastic therapy. Currently, the vast majority of cancer patients will receive combinations of multiple drugs in treatment regimens such as cyclophosphamide, hydroxydaunorubicin, oncovin and prednisone/prednisolone (CHOP); folinic acid, 5-FU and oxaliplatin (FOLFOX); or folinic acid, 5-FU, and irinotecan (FOLFIRI). In addition, chemotherapy can be combined with other treatment modalities such as RT, kinase inhibitors and surgery.

Based on technological advancements in the last decades it became possible to extensively screen cancer cells for specific vulnerabilities aiming at the development of novel, more specific therapeutic options. However, during the treatment course with these therapies, the tumor cells can quickly develop escape strategies to withstand the applied mono-treatments. Some examples of targeted therapies are growth factor receptor inhibitors (e.g. cetuximab) or kinase inhibitors (BRAF or MEK inhibitors) that downregulate hyperactive pathways leading to reduced cell growth. Here, intrinsic or acquired resistance leads to therapy failure if these molecules are applied as monotreatment [222–224]. In order to establish a successful combination therapy, first a cancer driving molecule needs to be identified as primary target. Second, knowledge of the molecular network around this first molecule needs to be established in order to find possible molecules as targets for combination treatments. As discussed in this review, MACC1 is such a promising target. MACC1 fuels cancer progression and metastasis formation leading to bad prognosis. Moreover, MACC1 gene expression can be inhibited by different small molecules.

In this context, statins inhibit MACC1 gene expression, leading to reduced cell motility in vitro and less metastasis formation in vivo due to reduced MACC1 production. Importantly, patients that use statins for the treatment of high cholesterol levels have a strongly reduced risk to develop cancer. In addition, the statins might inhibit farnesyl-transferases that are needed for RAS activity. MACC1 was shown to increase the activity of the RAS-RAF-MEK-ERK pathway thereby driving tumor progression [49]. This finding together with inhibitor studies—including clinically approved molecules (AZD6244, selumetinib; GSK1120212, trametinib)—pointed to an intimate connection of the MAP kinase pathway and MACC1. Indeed, MACC1 requires MEK1 activity to exert its full effectivity. This directly led to the idea of combining statins with MEK1 inhibitors. Via this combinational approach, the MACC1 function would be hit twice: the gene expression is reduced by statins, the remaining MACC1 protein is not fully activated due to inhibition of MEK1. Combined application of these two drug classes exhibited synergistic effect [49].

Furthermore, various members of the S100 protein family, including S100A4—a key metastatic molecule—can be induced by MACC1. The increased S100A4 gene expression was shown in vitro*, *in vivo—in genetically engineered mice—and in patient cohorts via transcriptome analysis. S100A4 is described as a direct target of the Wnt/β-catenin pathway [225]. This led to the identification of niclosamide as S100A4 inhibitor that acts via the β-catenin/TCF4 transcription activating complex [226]. The efficacy of niclosamide on cell motility in vitro and metastasis formation in vivo allowed for a first clinical phase II trial analyzing safety and efficacy in patients with metachronous or synchronous metastases of a CRC progressing after therapy [227]. Importantly, patients with a high gene expression of S100A4 and MACC1 showed the poorest prognosis. This prompted the testing of a therapeutic combination of statins (inhibiting MACC1) and niclosamide (inhibiting S100A4). Hitting the MACC1 function twice, at the gene expressions level by the statins and on the executing level via S100A4 using niclosamide, generated a strong synergistic effect by the combined treatment in vitro. This directly translated to the in vivo application, demonstrating effective metastasis restriction of more than 90% in murine models using human equivalent doses [92].

To replace the repositioned drug class of statins by a novel potentially superior molecule, a large high-throughput screen targeting the MACC1 gene expression was performed. Among 118.500 compounds, tetrazolo-pyridazine based small molecules were identified as the most promising primary hits. After structure–activity relationship (SAR) experiments, most effective compounds were validated for their ability to reduce MACC1 gene expression and cell motility in vitro and metastasis formation in vivo. Unlike the statins, the newly identified compounds act via the NF-κB pathway [119]. Since it was shown, that the MACC1 gene expression can be induced via TNFα/NF-κB [118] the combination of the new compound and niclosamide was tested in vitro. This testing revealed a synergistic efficacy of the novel compounds targeting MACC1 and niclosamide as an effective S100A4-inhibitor.

As discussed above, combined inhibition of different pathways that drive MACC1 gene expression and an additional target linked to MACC1, such as MEK1 or S100A4, can elicit synergistic effects. Therefore, it is to be expected, that further, novel combinations will show superior efficacy compared to the respective monotherapies. Here, all targets discussed above, e.g. miRNAs, antisense RNAs, SMIs and biologicals interfering in MACC1 expression and/or function as well as molecules targeting upstream or downstream targets and regulators of MACC1, might be explored for their synergistic efficacy. Of course, these combinations are not limited to two molecules. Combination regimens could be further extended if toxicity is within an acceptable range. In fact, drugs might exert higher efficacy in presence of other substances, which would allow dose reduction of individual drugs. For example, MACC1 induces resistance to chemotherapy by increasing ABCB1 gene expression. If MACC1 is downregulated, ABCB1 decreased in parallel. This might re-sensitize cancer cells towards chemotherapeutic drugs, which are originally restricted in use by the activity of ABCB1. Taken together, as a causal protein in cancer progression and metastasis formation, MACC1 holds a great promise not only as a valuable prognostic and predictive biomarker but simultaneously as a promising therapeutic target. An overview on molecules and therapeutic agents affecting MACC1 expression can be found in Table 1.

**Table 1:** MACC1 expression-affecting molecules

Molecule	Name	Entities	MoA and Outcome	References
**miRNA**	miR-15a	Cervical	• Bind to MACC1 transcript, leading to its degradation • MACC1 expression ↓	[228]
	miR-18a-5p	hepatocellular		[194]
	miR-18b-5p	hepatocellular		[194]
	miR-34a	hepatocellular, cervical squamous		[196, 201]
	miR-125a-5p	hepatocellular		[196]
	miR-126-5p	endometrial		[207]
	miR-145	hepatocellular, gastric, colorectal, nasopharyngeal,		[138, 141, 143, 194, 198]
	miR-145-3p	breast		[198]
	miR-145-5p	colorectal, gastric		[143, 189]
	miR-186-5p	osteosarcoma		[229]
	miR-195-5p	hepatocellular		[197]
	miR-320a	colorectal, gastric		[191, 230]
	miR-330-5p	colorectal		[193]
	miR-384	breast		[198]
	miR-497-5p	breast		[173]
	miR-605-3p	NSC lung		[207]
	miR-638	gastric		[231]
	miR-877	cervical		[232]
	miR-940	colorectal		[188]
	miR-1236-3p	colorectal		[188]
	miR-1304-5p	NSC lung		[206]
	miR-6867-5p	brain microvascular endothelial		[233]
	miR-141, miR-143, miR-200a, miR-218, miR-338-3p, miR-433, miR-485, miR-497, miR-547-5p, miR-590-3p, miR-598, miR-944	gastric, hepatocellular, colorectal, cervical, ovarian, Melanoma, glioma, gastric, breast, glioblastoma, nasopharyngeal, colorectal, NSC lung		Previously reviewed in [5]
**LncRNA**	LncRNA MACC1-As1	Gastric, glioma, human brain microvascular endothelial, lung, hepatocellular, breast, pancreatic, gastric, nasopharyngeal, esophageal squamous, osteosarcoma, pancreatic	• Suppresses various miRs such as miR-145, miR-384 • MACC1 expression ↑	[138, 141, 143, 172, 199, 203, 204, 219, 233–235]
	LncRNA-HSD17B11-1:1	Colorectal	• Suppresses miRNA expression • MACC1 expression ↑	[187]
	LncRNA FGD5-AS1	NSC lung, breast		[205]
	LINC01703	NSC lung		[207]
	LncRNA-CYTOR	Colorectal		[208]
	LncRNA ZFAS1	Colon adenocarcinoma		[148]
	LncRNA HCP5	Cervical		[228]
**Kinase inhibitors**	Crizotinib	Glioblastoma	• MACC1-induced migration, invasion, and elastic modulus ↓	[103, 213]
	MEK inhibitors	Colorectal	• Targeting MEK1 with inhibitors reduced MACC1 tyrosine phosphorylation and restricts MACC1-induced metastasis formation in vivo	[49]
	MET inhibitors	Pancreatic	• Reduction in MET activity leads to reduced MACC1-mediated VEGF-C/VEGF-D expression	[102]
**Transcriptional inhibitors**	Statins	Colorectal	• Block the DNA binding groove of Sp1 and c-Jun • MACC1 expression ↓	[41, 92, 147, 210]
	Tetrazolo-pyridazine compounds	Colorectal, esophageal, pancreatic, breast	• Reduces transcriptional activity of MACC1 promoter via NF-κB • MACC1 expression ↓	[119]
**Natural compounds**	Saffron	Colorectal	• Delays cell cycle progression at G2/M-phase • DCLK1 expression ↓ leading to MACC1 expression ↓	[132]
	Curcumin	Colorectal	• MACC1 expression ↓	[216]
	Nor-/Cantharidin	Colorectal	• MACC1 expression ↓	[217]
**Others**	Adalimumab	Colorectal	• Blocks TNF-α signaling • MACC1 expression ↓	[118]
	Parecoxib	Colorectal	• MACC1, c-Met, p-Akt, p-ERK expression ↓	[214]
	Verapamil	Colorectal	• MACC1 expression ↓	[177]
	Mitomycin	Colorectal	• Abolishes MACC1-associated effect of proliferation on collective migration speed	[220]

**Fig. 4:**
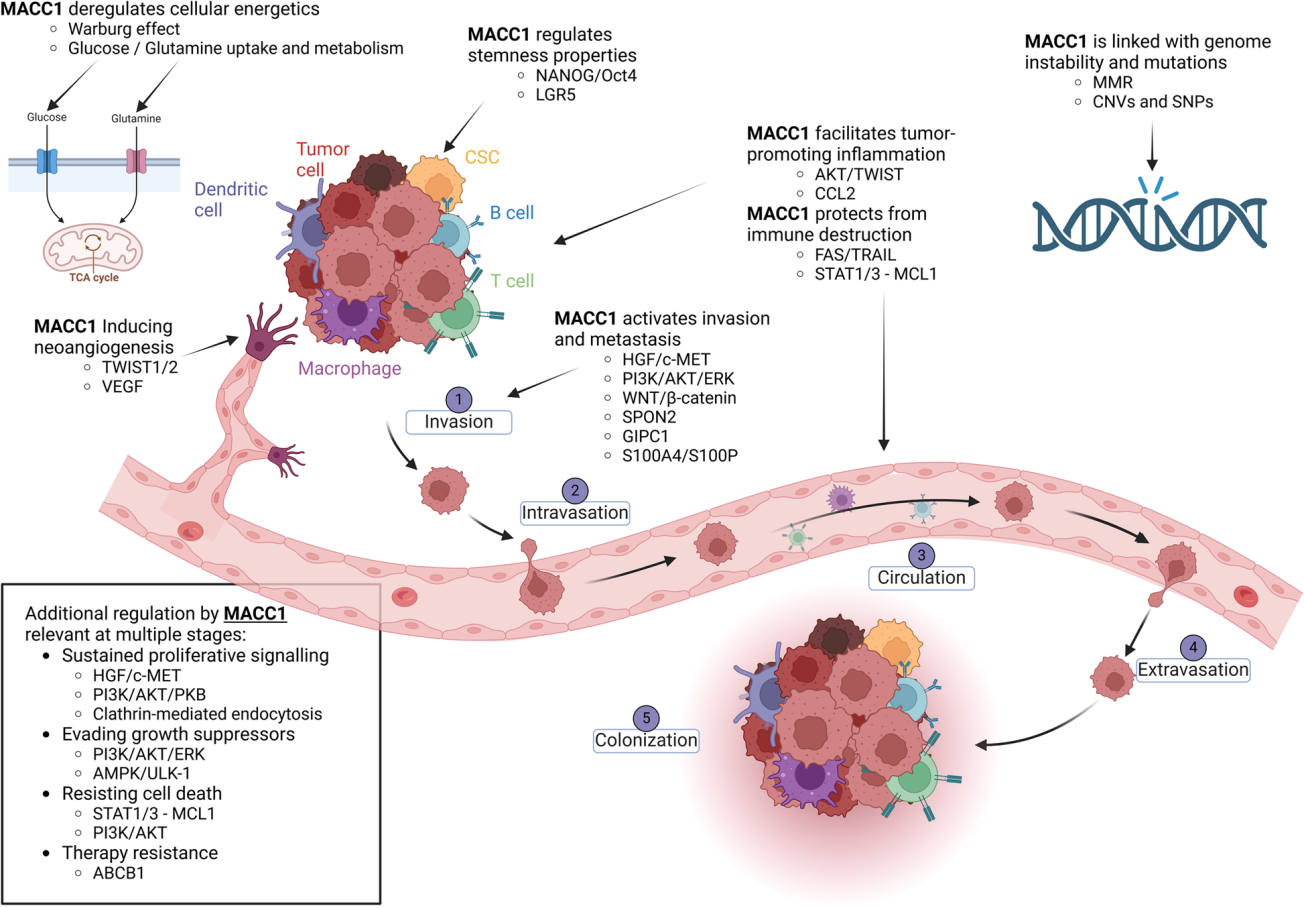
MACC1 affects the metastatic cascade at various steps. MACC1 serves as a master regulator of metastasis, directly or indirectly regulating properties of cancer cells that contribute to metastatic dissemination. Created with BioRender

Taken together, in this review we could highlight the importance of MACC1 in a clinical context as a predictive and prognostic factor in many cancer entities. We explored the interactions and regulations of MACC1 itself and within its´ signaling networks. Functionally, we highlighted the relevance of MACC1 for cancer properties such as stemness, immune evasion and therapy resistance. Lastly, we elaborated on intervention strategies targeting MACC1 and the potential of adding these approaches to the armamentarium of cancer therapies, either alone or in combinatorial regimes. A graphical representation of various factors of the metastatic cascade which are affected by MACC1 is shown in Fig. 4.

## Concluding remarks

Despite the new clinical and biological advancements in MACC1 research, numerous questions remain unanswered. For instance, the issue of the normal physiological role of MACC1 in tissue and blood of healthy individuals where MACC1 is hardly detectable in most of these samples, its involvement in stemness, in developmental processes, or its role in the context of obesity. This is also linked to the question, if MACC1 might play a role in other, non-cancerous diseases, such as Schwannoma, depression and hypertension.

Another under-investigated point is the role of MACC1 in the so-called MACC1 network. Since MACC1 mutations are rare, we mainly have to concentrate on its expression status and expression regulation. This provokes questions such as: under which physiological conditions is MACC1 epigenetically altered; is the MACC1 expression regulated by other genes; does MACC1 regulate other genes (transcriptionally); is MACC1 post-translationally modified; is MACC1 binding to protein–protein-binding partners (if so, to which partners) and/or is MACC1 potentially secreted for unknown extracellular functions? All these questions should be answered in the context of respective organs and tissues or depending on developmental stages.

One important aspect still to understand in more detail is how MACC1 induces cell migration/motility, which is a key biological feature of this gene/protein. Key molecular determinants of the MACC1 mediated metastatic switch are not yet defined but are necessary to contribute to more specific, targeted interventions hindering the development of metastases. Although there is novel information on repurposed drugs and novel compounds for MACC1 driven metastasis intervention, knowledge on their combinatorial use with conventional treatment, especially with immunological treatment strategies, is limited. Furthermore, novel therapeutic strategies should be implemented such as MACC1 targeting proteolysis targeting chimeras (PROTACs) or other protein degradation tools. And very importantly, it should be explored in detail which intervention strategy is useful to interfere at which specific stage of the metastasis evolution. All of these endeavors certainly require clinical trials, but we have to face these challenges to successfully fight metastatic cancer—here at the level of MACC1-induced metastasis as a new therapeutic paradigm.

## Data Availability

No datasets were generated or analysed during the current study.
